# Editorial: Caregiver burden in Alzheimer's and other chronic conditions

**DOI:** 10.3389/fpsyg.2026.2007129

**Published:** 2026-10-20

**Authors:** Erika Melonashi, Margarida Vilaça, M. Graça Pereira

**Affiliations:** 1 Department of Psychology, Education and Sports, European University of Tirana, Tirana, Albania; 2 Faculty of Psychology and Educational Sciences, University of Coimbra, Coimbra, Portugal; 3 School of Psychology, University of Minho, Braga, Portugal

**Keywords:** caregiver burden, caregiver interventions, chronic illness, coping, dementia, health policy, psychological resilience

Most individuals with chronic illnesses rely on unpaid, untrained caregivers who bear immense health, financial, and emotional costs. Decades of research confirm caregiving is burdensome, but the critical question remains: why do similar demands yield such divergent outcomes, and how can we shift them? The 13 contributions in this Research Topic examine conditions from Alzheimer's and brain injuries to schizophrenia and autism. Findings deliver a unified message: caregiver burden is not uniform, resilience is not static, and support systems designed for population averages often fail those who need them most.

## Mapping the caregiving burden

Four studies highlight the complexity of caregiver burden among dementia caregivers by highlighting the need to consider local level variables, atypical cases, dementia status, and associated syndromes. Although the availability of nationally representative data has substantially advanced our understanding of the health implications of dementia caregiving, less is known about the prevalence, caregiving context, and wellbeing of dementia caregivers at the local level. Gaugler et al. analyzed 2021–2022 Behavioral Risk Factor Surveillance System data across 47 states and within five specific states reporting substantial heterogeneity at state level. These findings clearly suggest that national averages used to inform policies may misdirect home- and community-based services, if local level variables are not considered.

Further expanding this scope, Mize et al. examine complex, atypical caregiving roles—such as minor children caring for parents with young-onset dementia, aging parents supporting adult children with neurodegenerative conditions, or caregivers experiencing their own cognitive decline. Younger caregivers often struggle due to limited life experience, while aging parents face physical limitations that hinder their ability to meet growing care demands. Identifying and developing targeted resources for these complex, underserved populations is critical to addressing gaps left by traditional, broad-scale healthcare planning.

Shubair, drawing on a sample of 2,652 respondents from the National Health and Aging Trends Study linked to the National Study of Caregiving, shows that income disparities and caregiving-related strain is graded by dementia status. Caregivers of older adults with probable and possible dementia reported the higher physical, emotional, and financial strain as compared to caregivers of non-dementia individuals. Moreover, Su et al. reported that caregivers of patients with dementia with Lewy bodies who also had Pisa syndrome, experienced greater distress and burden related to delusion, hallucination, anxiety, aberrant motor behavior, and sleep disturbance. These results underscore the importance of comprehensive management strategies considering dementia status and presence of other syndromes.

## Protective and risk factors

Six studies provide convergent evidence on protective and risk factors underlying caregiver outcomes, identifying modifiable psychological or physiological factors that mediate the relationship between objective caregiving demands and outcomes.

Nichols and Laine applied latent class analysis to 1,026 US informal caregivers, deriving five motivational profiles which moderated the associations among caregiving stressors, resources, and wellbeing. Motivation profiles significantly shaped how caregiving demands and resources were associated with caregiver wellbeing, with duty-, affective-, obligation-, cultural-, and situationally motivated caregivers exhibiting distinct patterns of distress, burnout, and resilience. Resilience was consistently protective, whereas social support showed variable and sometimes paradoxical effects across motivation profiles.

In a 12-month, three-wave study of 130 Portuguese family caregivers of people with Alzheimer's disease, Brito et al. found that physical quality of life and health behaviors initially declined before exhibiting a recovery trend. Conversely, mental quality of life decreased steadily without rebounding. Psychological distress indirectly influenced caregiver burden and physical quality of life through forgiveness as a mediator. These findings highlight that post-diagnosis adaptation is domain-specific, showing how physical recovery can obscure persistent mental health decline.

Examining informal family caregivers of patients with schizophrenia, Liu Y. et al. found that hope partially mediates the relationship between anxiety and preparedness (~25% of the total effect), establishing that affective states directly gate whether practical skills feel usable. Song et al. studied 85 parent–child dyads at two autism rehabilitation centers in central China, concluding that children's light-intensity physical activity is positively associated with parents' own activity levels and with the psychological and environmental domains of parental quality of life. Viewed through the lens of reciprocal determinism, the findings demonstrate the dynamic, bi-directional interplay among child behavior, parental psychological health, and the broader family environment.

Extending this framework to formal healthcare settings, Zhang et al. modeled data from 1,630 hemodialysis nurses, revealing that insomnia and fatigue mediate the pathway from self-efficacy and resilience to overall mental health status, highlighting rest as a critical biological leverage point in professional workforce strain. Complementing these quantitative models, Deng et al.'s qualitative meta-synthesis on the experiences of family caregivers of individuals with cognitive impairment grounds these mechanisms in lived experience, consolidating 74 findings across four core themes: gaps in disease literacy, negative caregiving experiences, network relational challenges, and the necessity of adaptive coping. Synthesizing these perspectives reveals that sustaining both informal and formal caregivers requires a dual approach: mitigating physiological depletion like sleep disruption while providing the psychological and educational scaffolding necessary to sustain hope and functional preparedness.

## Intervention and policy gaps

Two meta-analyses focus on caregiver interventions. Ting and Ahmed identified thirteen randomized trials of caregiver interventions in adult traumatic brain injury, finding significant improvement in burden and a smaller but significant reduction in psychological distress—tempered by high risk of bias and marked heterogeneity in studies. Liu F. et al. pooled 25 trials involving 2,425 caregivers of chronic dyspnea patients, reporting a moderate reduction in burden. Authors reported substantial heterogeneity, with the largest effect in social burden, and significant improvements in stress, depression, and family function—but no significant change in anxiety or quality of life. These two studies suggest that interventions reliably shift burden and mood, yet leave quality of life largely untouched, and heterogeneity quite high. The most plausible reading is that we are averaging across interventions that work for different people through different mechanisms—precisely what the motivational profiles of Nichols and Laine would predict.

Li et al. make the case of how policies might serve as binding constrains by analyzing the systemic case from China, where dementia affects more than 16.9 million people and is projected to reach nearly 49 million by 2050, with roughly 80% of patients cared for at home. Filial obligation combined with one-child policy demographics leaves many caregivers without rotation or respite, and dementia care remains largely outside long-term care insurance. Their recommendations, including funding for caregiver mental health services, adoption of WHO iSupport, insurance reimbursement, tax credits and allowances, expanded respite, and assistive technology, are concrete and costed against an existing national action plan. Gaugler et al. and Li et al. bookend the policy problem neatly. One shows that even a mature national surveillance infrastructure produces data too coarse for local planning; the other shows what follows when policy attends to the patient and overlooks the caregiver.

## Conclusions

Research demonstrates that caregiver burden is a complex, condition-specific experience requiring tailored support rather than broad, generalized policies. Across neurodegenerative diseases, psychiatric conditions, brain injuries, and high-stress clinical roles like hemodialysis nursing, caregivers face severe physical, psychological, social, and financial strain.

Internal protective factors, including resilience, hope, self-efficacy, and physical activity, frequently mediate or moderate against anxiety, burnout, and physical decline. While targeted psychosocial, educational, and technological interventions show strong potential to alleviate distress, a significant disconnection remains between rigid, national-level healthcare planning and localized, lived realities.

To effectively sustain caregiver wellbeing, healthcare systems, researchers, and policymakers need to reject “one-size-fits-all” approaches. Instead, future policy frameworks must integrate informal caregivers into resource allocations by combining local data, targeted psychological scaffolding, and concrete structural reforms.

